# Association between glycation gap and impaired cardiorespiratory fitness: evidence from American adults

**DOI:** 10.1186/s12872-025-04578-y

**Published:** 2025-02-20

**Authors:** Min Fei, Bo Wu, Jiabin Tu, Hongkui Chen, Yansong Guo

**Affiliations:** 1 Department of Cardiology, Fujian Provincial Hospital, Shengli Clinical Medical College of Fujian Medical University, Fuzhou University Affiliated Provincial Hospital, Fuzhou, 355000 China; 2 Department of Cardiology, Longyan First Affiliated Hospital of Fujian Medical University, Longyan, 364000 China

**Keywords:** Glycation Gap, Cardiorespiratory Fitness, NHANES, Cardiovascular Health, HbA1c

## Abstract

**Background:**

Cardiorespiratory fitness (CRF) is a critical indicator of overall health, while the glycation gap (G-Gap) emerges as a potential novel biomarker for metabolic and cardiovascular risk assessment. However, the relationship between G-Gap and CRF remains incompletely understood.

**Objective:**

To investigate the association between glycation gap and impaired CRF, and evaluate its potential as an early health risk indicator.

**Methods:**

Using data from the National Health and Nutrition Examination Survey (NHANES, 1999–2004), we conducted a comprehensive analysis of 3,818 adult participants. G-Gap was calculated by standardizing glycated albumin (GA) and glycated hemoglobin (HbA1c) levels, comparing actual and predicted HbA1c values. Cardiorespiratory fitness was assessed through maximal oxygen uptake (VO2 max), with impaired CRF defined as performance below the 20th percentile for gender and age-specific thresholds. Multivariate logistic regression models were employed, adjusting for demographic characteristics, laboratory parameters, and potential confounding factors.

**Results:**

In unadjusted models, For every 1 increase in G-Gap as a continuous variable, the chance of CRF damage increased by 65% (OR 1.65, 95% CI 1.29–2.11). After comprehensive covariate adjustment, the association remained statistically significant, with odds ratios of 1.87 (95% CI 1.41–2.49) in partially adjusted and 1.41 (95% CI 1.01–1.98) in fully adjusted models. Quartile analysis revealed significantly higher risks of impaired CRF in the third and fourth G-Gap quartiles compared to the first quartile.

**Conclusions:**

This study demonstrates an association between higher G-Gap values and an increased likelihood of impaired CRF.

**Supplementary Information:**

The online version contains supplementary material available at 10.1186/s12872-025-04578-y.

## Introduction

Cardiorespiratory fitness (CRF) reflects an individual's capacity to transport and utilize oxygen during prolonged physical exertion. It reflects the integrated efficiency of the cardiovascular, respiratory and muscular systems in oxygen utilization [1]. CRF is widely regarded as a key indicator of overall health and physical performance. Research by Kokkinos et al. has demonstrated a significant association between CRF and mortality risk [2]. Furthermore, studies conducted by Lang et al. indicate that improving CRF levels can reduce the risk of developing various chronic diseases [3]. While impaired CRF is not currently defined as a disease, it is generally considered to be a precursor to many metabolic diseases.

Given the importance of CRF to health, researchers have begun to explore various factors influencing CRF. In this context, the potential relationship between blood glucose control and CRF has garnered widespread attention. Glycated hemoglobin (HbA1c), as the standard indicator for assessing long-term blood glucose control, has become an important biomarker for investigating the association between blood glucose control and CRF. Some studies have found a positive association between HbA1c levels and impaired CRF [4, 5].

However, recent evidence suggests that HbA1c levels may not always accurately reflect average blood glucose levels for all individuals due to various factors, including the activity of glycolysis and deglycation enzymes, as well as red blood cell lifespan. This discrepancy has led to the introduction of the concept of the "Glycation Gap" (G-Gap) [6]. The G-Gap reflects the difference between the actual measured HbA1c and the HbA1c predicted based on fructosamine/glycated albumin (GA) levels. The G-Gap not only serves as an alternative indicator for assessing blood glucose control but also quantifies an individual's glycation tendency, potentially indicating susceptibility to glycation-related damage [7, 8]. Studies have shown that the G-Gap is associated with various health risks [9–11]. Under prolonged exposure to a high-glucose environment, the N-terminal amino acid residues of hemoglobin can be non-enzymatically glycosylated by glucose, which alters the protein structure and membrane function of red blood cells, thus affecting oxygen transport [12]. Moreover, glycation products not only exist in red blood cells but also bind to vascular walls or circulating proteins, triggering a series of inflammatory responses and causing endothelial dysfunction. Such microcirculatory disorders can lead to insufficient tissue oxygen supply and further increase the burden on the cardiovascular system [13]. Understanding the relationship between G-Gap and CRF may aid in early health risk identification and the development of personalized prevention strategies. However, despite the increasing importance of G-Gap in health risk assessment, there is currently a lack of research exploring the association between G-Gap and CRF.

The purpose of this study is to analyze the association between G-Gap and impaired CRF using multivariate regression analysis through the NHANES database, to provide an important basis for early health risk identification and personalized prevention strategies, and to provide new insights into the complex relationship between glucose metabolism and cardiorespiratory function.

## Methods

### Study population

**Fig. 1:**
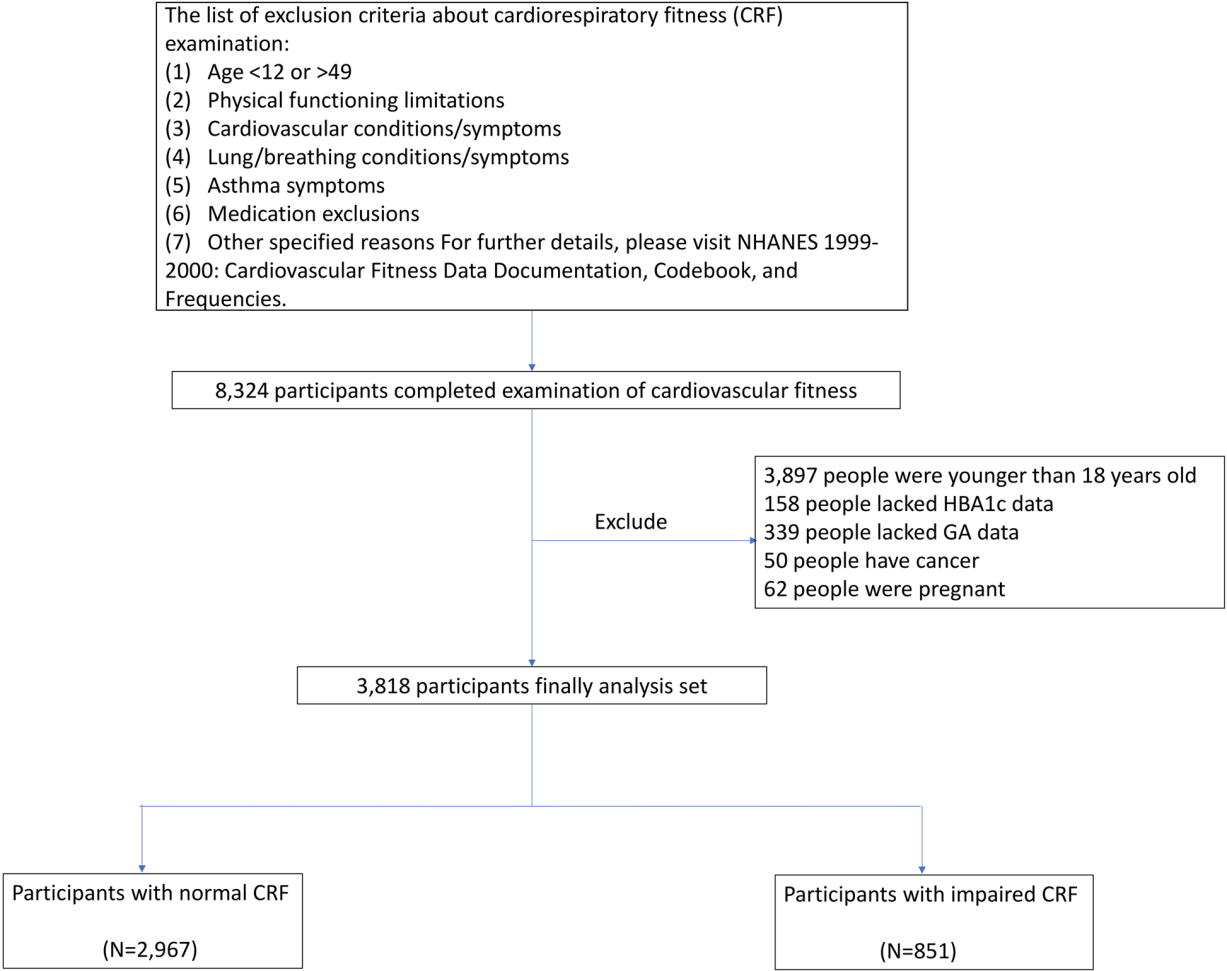
Flow chart

The data for this study were drawn from the National Health and Nutrition Examination Survey (NHANES) surveys conducted between 1999–2004, as CRF measurements were only collected during those two cycles. Initially, 8,324 participants completed the CRF examination. We applied the following exclusion criteria:1. Age < 18 years (*n* = 3,897); 2. Missing HbA1c data (*n* = 158); 3. Missing GA data (*n* = 339); 4. Cancer diagnosis (*n* = 50); 5. Pregnancy (*n* = 62). After applying these exclusion criteria, the final analytical sample comprised 3,818 participants. These participants were further categorized into two groups based on their CRF status: Normal CRF group (*n* = 2,967), Impaired CRF group (*n* = 851). Figure 1 shows the detailed participant screening process.

It should be noted that the original CRF examination had additional exclusion criteria, including physical limitations, cardiovascular diseases/symptoms, pulmonary/respiratory diseases/symptoms, asthma symptoms, medication exclusions, and other specific reasons. The specific criteria are outlined in the supplementary materials (Table S1).

### Definition of G-Gap

The G-Gap was calculated using GA to assess the difference between measured and predicted HbA1c values. Initially, both GA and HbA1c levels were measured for each participant. The mean and standard deviation (SD) of GA and HbA1c were then calculated. The GA value was standardized by converting it to its standard normal deviate (SND) using the formula: SND_GA = (GA—Mean_GA) / SD_GA. Subsequently, this SND of GA was used to calculate the GA-derived HbA1c equivalent (G_HbA1c) using the formula: G_HbA1c = (SND_GA × SD_HbA1c) + Mean_HbA1c. Finally, the G-Gap was determined by calculating the difference between the actual HbA1c and the predicted G_HbA1c: G-Gap = HbA1c—G_HbA1c. To facilitate trend analysis (P for trend) and achieve better risk stratification, participants were divided into four groups based on the quartiles of the G-Gap values: Q1 (G-Gap < −0.2331), Q2 (−0.2331 ≤ G-Gap < −0.0058), Q3 (−0.0058 ≤ G-Gap < 0.2253) and Q4 (G-Gap ≥ 0.2253).

### Definition of impaired CRF

In the NHANES protocol, CRF assessment involves a treadmill exercise test where maximal oxygen uptake (VO2 max) serves as the key measurement. The test protocol requires participants to exercise at increasing intensities by adjusting treadmill speed and grade until reaching their target heart rate. Since oxygen consumption and heart rate show a direct correlation during exercise, researchers can estimate VO2 max by monitoring heart rate responses at predefined submaximal exercise levels.

The classification of CRF status relies on gender- and age-specific VO2 max thresholds established by the Aerobics Center Longitudinal Study (ACLS) [14]. NHANES utilized a submaximal exercise test methodology that estimates VO2max based on the linear relationship between heart rate and oxygen consumption during exercise. Here's the specific calculation method: Participants complete two stages of submaximal exercise. Uses the equation: Estimated VO2max = (PMHR (Predicted Maximum Heart Rate)—Intercept) / Slope. Where PMHR = 220—Age. Slope and intercept are calculated using heart rates and oxygen consumption values from both stages. This protocol has been widely adopted in practice due to its convenience and cost-effectiveness. Wang et al. demonstrated a correlation coefficient of 0.79 between estimated VO₂ max derived from the NHANES protocol and directly measured VO₂ max values [15]. Participants are categorized into three fitness levels: those with estimated VO2 max values falling below the 20th percentile of their age-gender group are considered to have low fitness, while values between the 20th and 59th percentiles indicate moderate fitness. High fitness is assigned to individuals whose VO2 max reaches or exceeds the 60th percentile. For the purposes of this research, impaired CRF was defined as having low fitness status. Further methodological details are available in the NHANES CRF documentation (https://wwwn.cdc.gov/Nchs/Data/Nhanes/Public/1999/DataFiles/cvx.htm#CVDESVO2).

### Confounding variable

Age, sex, race/ethnicity, smoking status, alcohol consumption, education level, and poverty income ratio (PIR) were self-reported by participants. Diagnostic criteria for comorbidities are detailed in supplementary materials (Table S2). All blood samples were collected by NHANES staff and subsequently analyzed in laboratories. Detailed information on laboratory equipment and protocols can be found in the manuals available on the official NHANES website. Body Mass Index (BMI) was calculated using height and weight measurements obtained by NHANES official staff.

### Statistical analysis

NHANES employs a complex survey design, necessitating the use of sampling weights during data analysis to ensure the results are representative of the entire U.S. population. These weights are provided in the official NHANES dataset, so all statistical analyses in this study were conducted using weighted calculations. For the baseline characteristics, continuous variables are presented as means with standard errors to reflect the representativeness of the sample, while categorical variables are displayed as counts with weighted percentages. Appropriate statistical tests, such as analysis of variance (ANOVA) for continuous variables and chi-square tests for categorical variables, were used to assess between-group differences.

Logistic regression was initially employed to identify confounding variables associated with impaired CRF. Variables that remained significantly associated with impaired CRF after adjusting for age, sex, and race were defined as important confounding factors. To enhance the robustness of our results, we used three regression models to analyze the relationship between G-Gap and impaired CRF. Model 1 was unadjusted. Model 2 adjusted for basic demographic variables: sex, age, and race. Model 3 further included the important confounding variables identified earlier. Restricted cubic splines were used to visualize the results of Model 3. Likelihood ratio tests were conducted to explore potential threshold effects. To validate the consistency of G-Gap's effects across different populations, subgroup analyses were performed, with results visualized using forest plots and restricted cubic splines. Lastly, we analyzed the associations of GA and HbA1c with impaired CRF.

All statistical analyses were performed using R studio 4.3.3. Bilateral *P* value < 0.05 could be considered statistically significant.

## Results

### Participant characteristics

A total of 3,818 participants were included in this study. The mean age of the participants was 32.7 years old. Participants with impaired CRF were significantly younger (30.3 years old) compared to those with normal CRF (33.1 years old) (*P* < 0.001). The study population comprised 46.7% females and 53.3% males, with a significant difference in gender distribution between the normal and impaired CRF groups (*P* = 0.012). Smoking and alcohol use were reported in 56.7% and 80.6% of participants, respectively, with no significant differences between CRF groups (*P* = 0.101 and *P* = 0.164, respectively). Education levels varied, with 61.0% having more than a high school education, 24.88% with high school education, and 14.2% with less than high school education. The impaired CRF group tended to have lower education levels, although this difference was not statistically significant (*P* = 0.060). Participants with impaired CRF had significantly higher rates of obesity (37.3% vs. 21.5%, *P* < 0.001), hyperlipidemia (67.8% vs. 59.2%, *P* < 0.001), and DM (3.8% vs. 1.8%, *P* = 0.032) compared to those with normal CRF. Laboratory parameters, including HB, WBC, and PLT, showed statistically significant differences between the normal and impaired CRF groups (all *P* < 0.001). The detailed baseline characteristics of the study population were summarized in Table 1.

**Table 1:** Baseline characteristics of participants

variable	Total (*N* = 3,818)	Normal CRF (*N* = 2,967)	Impaired CRF (*N* = 851)	*P*-value
Age, years	32.7(0.2)	33.1(0.3)	30.3(0.5)	< 0.001
Gender, n (%)				0.012
Female	1,770(46.7)	1,316(45.6)	454(52.5)	
Male	2,048(53.3)	1,651(54.5)	397(47.5)	
Race, n (%)				< 0.001
Mexican American	1,080(9.9)	792(9.3)	288(12.4)	
Non-Hispanic Black	811(9.8)	586(8.8)	225(14.9)	
Non-Hispanic White	1,612(69.9)	1,347(72.0)	265(59.8)	
Other Race	315(10.4)	242(9.9)	73(13.0)	
Smoker, n (%)	1,205(56.7)	1,034(44.2)	171(39.0)	0.101
Alcohol user, n (%)	2,134(80.6)	1,801(81.4)	333(76.2)	0.164
Education, n (%)				0.060
< High school	640(14.2)	533(13.7)	107(17.0)	
High school	684(24.9)	562(24.4)	122(27.6)	
> High school	1,510(61.0)	1,272(62.0)	238(55.5)	
PIR	3.06(0.06)	3.13(0.06)	2.75(0.07)	< 0.001
Obesity, n (%)	916(24.2)	616(21.5)	300(37.3)	< 0.001
Hypertension, n (%)	416(12.2)	319(11.6)	97(15.1)	0.051
DM, n (%)	89(2.1)	66(1.8)	23(3.8)	0.032
CKD, n (%)	208(4.5)	158(4.7)	50(3.7)	0.249
Hyperlipidemia, n (%)	2,162(60.7)	1,656(59.2)	506(67.8)	< 0.001
HB, g/dl	14.64(0.05)	14.71(0.05)	14.34(0.10)	< 0.001
WBC,1000/ul	7.13(0.05)	7.05(0.06)	7.52(0.10)	< 0.001
PLT,1000/ul	270.03(1.37)	266.74(1.45)	286.04(3.60)	< 0.001
CRP, mg/dl	0.30(0.01)	0.28(0.01)	0.41(0.03)	< 0.001
HbA1c, %	5.21(0.01)	5.19(0.01)	5.30(0.03)	0.007
GA, %	13.30(0.14)	13.35(0.16)	13.07(0.15)	0.167
G-Gap, %	−0.02(0.02)	−0.04(0.02)	0.09(0.03)	< 0.001

*Abbreviation: DM* Diabetes mellitus, *CKD* Chronic kidney disease, *HB* Hemoglobin, *WBC* White blood cell, *PLT* Platelet, *PIR* Poverty income ratio, *CRP* C-reactive protein, *HbA1c* Glycated hemoglobin, *GA* Glycated albumin, *G-Gap* Glycation gap

### Important confounding variables

After adjusting for age gender and race, the important blood parameters that remained significantly associated with impaired CRF in the logistic regression analysis were HB, WBC, and PLT, with OR, 95% CI, and *P*-values of (0.83, 0.74–0.93, *P* = 0.001), (1.11, 1.06–1.16, *P* < 0.001), and (1.00, 1.00–1.01, *P* < 0.001), respectively. Among the comorbidities, DM, hypertension, hyperlipidemia, and obesity were associated with impaired CRF, with OR, 95% CI, and *P*-values of (2.37, 1.18–4.79, *P* = 0.017), (1.63, 1.18–2.25, *P* = 0.004), (1.71, 1.39–2.17, *P* < 0.001), and (2.32, 1.83–2.94, *P* < 0.001), respectively. Other variables, including CRP, education level, smoking, alcohol consumption, and CKD, were not statistically significant (P > 0.05). Detailed information is shown in Table 2.

**Table 2:** Relationship between confounding variables and CRF impairment

Variable	OR (95% CI)	*P*-value
CRP	1.35(0.96,1.88)	0.079
HB	0.83(0.74,0.93)	0.001
WBC	1.11(1.06,1.16)	< 0.001
PLT	1.00(1.00,1.01)	< 0.001
PIR	0.95(0.89,1.01)	0.070
Education		
< High school	Ref	
High school	1.07(0.75,1.54)	0.690
> High school	0.85(0.60,1.22)	0.377
Smoke		
No	Ref	
Yes	0.87(0.67,1.12)	0.263
Alcohol.user		
No	Ref	
Yes	0.80(0.52,1.24)	0.308
DM		
No	Ref	
Yes	2.37(1.18,4.79)	0.017
Hypertension		
No	Ref	
Yes	1.63(1.18,2.25)	0.004
CKD		
No	Ref	
Yes	0.70(0.46,1.06)	0.087
Hyperlipidemia		
No	Ref	
Yes	1.71(1.39,2.17)	< 0.001
Obesity		
No	Ref	
Yes	2.32(1.83,2.94)	< 0.001

Adjusted by age, gender and race

### The relationship between G-Gap and impaired CRF

**Fig. 2:**
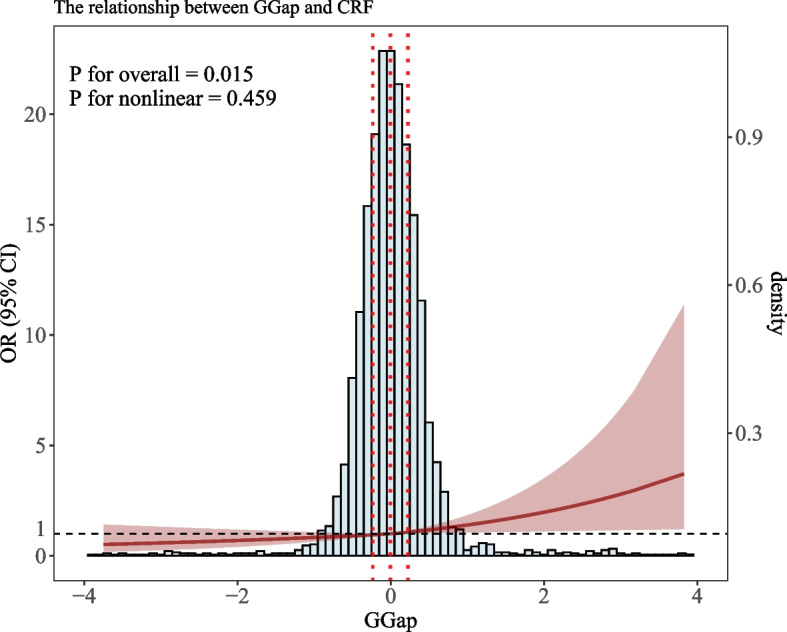
Dose–response relationship between G-Gap and impaired CRF

A significant positive association between G-Gap and impaired CRF was observed across all three models. In the unadjusted Model 1, every one increase in G-Gap was associated with an odds ratio (OR) of 1.65 (95% CI: 1.29–2.11, *P* < 0.001) for impaired CRF. This association strengthened in Model 2 after adjusting for demographic characteristics, with an OR of 1.87 (95% CI: 1.41–2.49, *P* < 0.001). In the fully adjusted Model 3, although slightly attenuated, the association remained significant with an OR of 1.41 (95% CI: 1.01–1.98, *P* = 0.046). Furthermore, the analysis revealed a relationship between quartiles of G-Gap (Q1,Q2, Q3, Q4) and the risk of impaired CRF. Across all three models, both Q3 and Q4 groups showed higher risks of impaired CRF compared to the reference group (Q1), with a significant dose–response relationship (all P for trend < 0.05). The details are shown in Table 3. Similar results were observed in the RCS analysis, further supporting the relationship between G-Gap and impaired CRF (Fig. 2).

**Table 3:** The association between G-Gap and CRF (weighted)

	Model 1		Model 2		Model 3	
	OR (95%CI)	*P*-value	OR (95%CI)	*P*-value	OR (95%CI)	*P*-value
**Continuous variables**						
G-Gap	1.65(1.29,2.11)	< 0.001	1.87(1.41,2.49)	< 0.001	1.41(1.01,1.98)	0.046
**Categorical variable**						
Q1 Group	Ref		Ref		Ref	
Q2 Group	1.29(0.99,1.69)	0.062	1.39(1.06,1.83)	0.020	1.22(0.92,1.61)	0.154
Q3 Group	1.54(1.15,2.08)	0.005	1.78(1.35,2.34)	< 0.001	1.38(1.01,1.88)	0.044
Q4 Group	1.88(1.41,2.51)	< 0.001	2.35(1.72,3.22)	< 0.001	1.47(1.05,2.04)	0.025
*P* for trend		< 0.001		< 0.001		0.027

Note: Q1 (G-Gap < −0.2331), Q2 (−0.2331 ≤ G-Gap < −0.0059), Q3 (−0.0059 ≤ G-Gap < 0.2253)and Q4 (G-Gap ≥ 0.2253)

Model 1: Unadjusted

Model 2: Adjusted by age, race, gender

Model 3: Adjusted by age, race, gender, HB, WBC, PLT, Obesity, DM, Hypertension, Hyperlipidemia

### The association between GA, HbA1c and CRF

In the three logistic regression models, GA showed no statistical association with impaired CRF. HbA1c was positively associated with impaired CRF in the unadjusted model (OR: 1.33, 95% CI: 1.08–1.65, *P* = 0.008). However, after adjusting for confounding factors, there was no significant statistical association between HbA1c and impaired CRF (Table 4).

**Table 4:** The association between GA, HbA1c and CRF (weighted)

	Model 1		Model 2		Model 3	
	OR (95%CI)	*P*-value	OR (95%CI)	*P*-value	OR (95%CI)	*P*-value
**Continuous variables**						
GA	0.97(0.92,1.04)	0.408	0.97(0.91,1.03)	0.307	0.97(0.92,1.03)	0.305
HbA1c	1.33(1.08,1.65)	0.008	1.42(1.12,1.79)	0.005	1.21(0.93,1.57)	0.151

Model 1: Unadjusted

Model 2: Adjusted by age, race, gender

Model 3: Adjusted by age, race, gender, HB, WBC, PLT, Obesity, DM, Hypertension, Hyperlipidemia

### Subgroup analysis

**Fig. 3:**
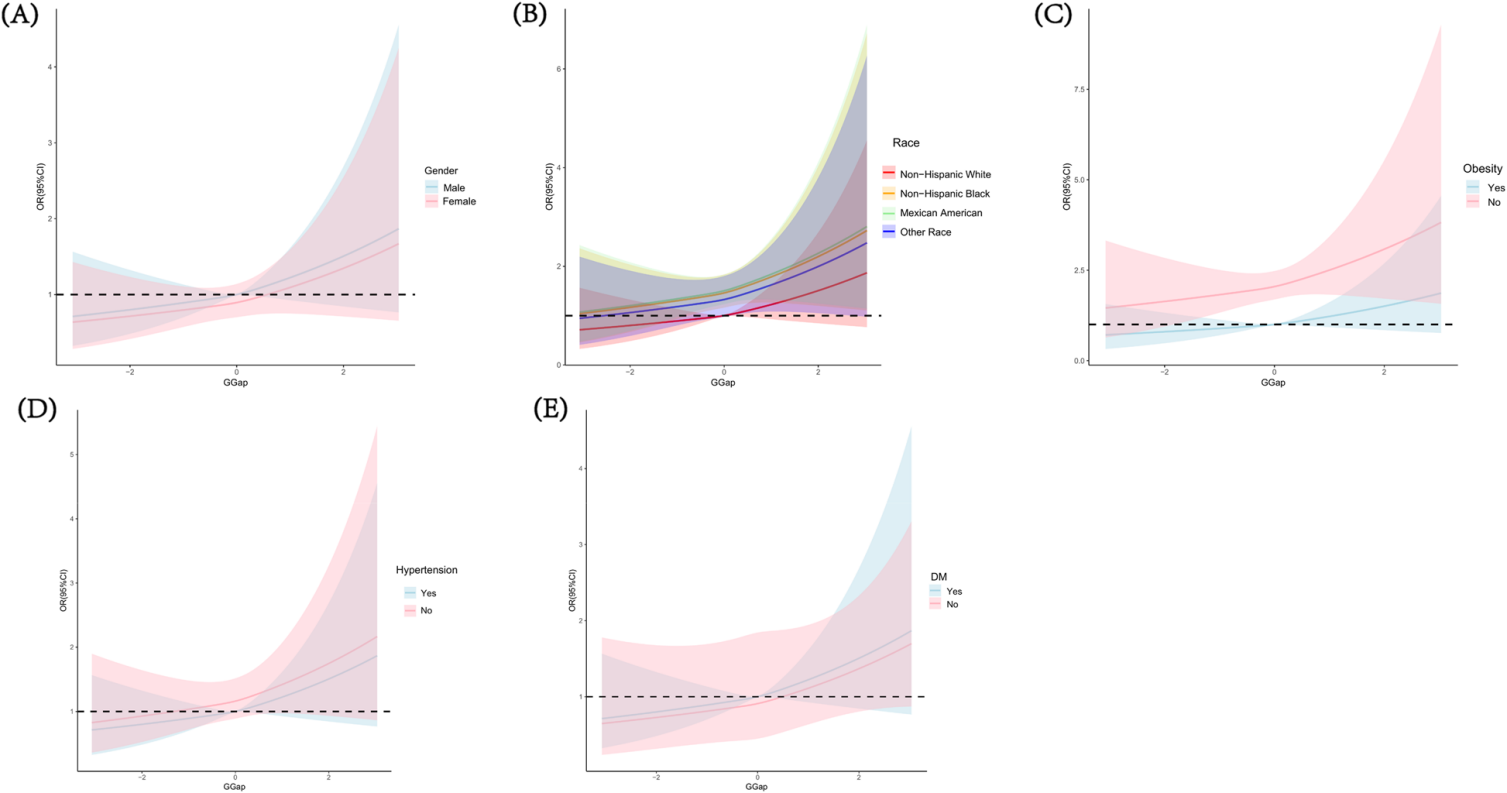
Dose–response relationship between G-Gap and CRF impairment in different subgroups

**Fig. 4:**
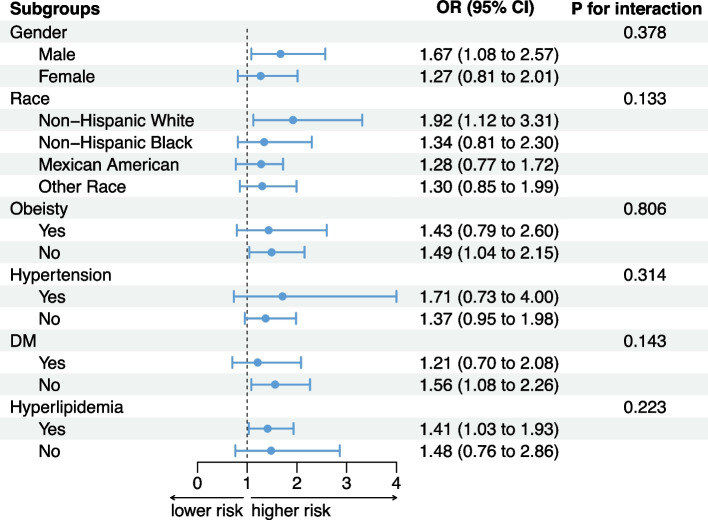
Subgroup Analysis of the Association Between G-Gap and CRF Impairment

After stratification by sex, race, obesity, hypertension, DM, and hyperlipidemia, the dose–response relationship between G-Gap and impaired CRF remained largely unchanged (Fig. 3). Moreover, no significant interactions were observed between the presence of comorbidities and G-Gap (Fig. 4).

### Supplementary analysis

In the threshold effect analysis, a potential threshold point for G-Gap was identified at −0.588. However, this threshold did not reach statistical significance (P for likelihood ratio test = 0.340, Table S3), suggesting a potentially continuous relationship between G-Gap and impaired CRF without a clear cut-off point.

## Discussion

This cross-sectional study analyzed data from 3,818 U.S. adults. The results showed that G-Gap was significantly and positively associated with impaired CRF. This association remained even after adjusting for age, sex, race, and other important confounding factors. RCS analysis revealed a dose–response relationship between G-Gap and impaired CRF, and this association was not significantly altered across different subgroups.

CRF is an important marker of overall health, reflecting the coordinated efficiency of the cardiovascular, respiratory, and muscular systems [16]. High CRF levels are associated with lower all-cause and CVD mortality, while impaired CRF is considered a precursor to many metabolic disorders [17–19].

The G-Gap reflects individual biological variations in the glycation process. Research has demonstrated that G-Gap may be associated with the development of DM-related complications, with particular significance in the progression of diabetic nephropathy [18, 20, 21]. Furthermore, research conducted by Wang et al. established that G-Gap is significantly associated with both CVD incidence and CVD mortality risk [22, 23]. Our findings demonstrate that elevated G-Gap values are associated with impaired CRF. This study provides the first direct investigation of the relationship between G-Gap and CRF, suggesting that G-Gap may be indicative of deteriorating cardiovascular health prior to the clinical manifestation of CVD.

When analyzing the associations of HbA1c and GA with impaired CRF, the results demonstrated no statistically significant association between either HbA1c or GA and impaired CRF. This finding suggests that G-Gap may provide unique information beyond that captured by HbA1c alone. Moreover, in this study, the proportion of patients with DM was relatively low, but after stratification in subgroup analysis, a strong association between G-Gap and impaired CRF was observed regardless of whether DM was present. Studies have confirmed that, even in individuals without DM, glycation level was an important component of cardiovascular health [24, 25]. This evidence further supports that G-Gap may be an independent predictor of impaired CRF. Relying solely on HbA1c levels may not fully identify the health risks posed by blood sugar changes. In individuals without DM who have normal HbA1c levels, a higher G-Gap is also associated with a decline in CRF. In addition, when stratifying according to variables such as race, although the insignificance of confidence interval and *p*-value is observed, the interaction *P*-value is not significant, and the OR value is always greater than 1. Therefore, the insignificance of the results after stratification may be affected by the sample size.

The positive association between higher G-Gap values and an increased likelihood of impaired CRF may be attributed to individuals with higher G-Gap being more susceptible to glycation-related damage, which affects the coordination and efficiency of cardiopulmonary function [26]. Studies have shown that the accumulation of Advanced Glycation End Products (AGEs) will lead to vascular stiffening and endothelial dysfunction, impairs myocardial tissue elasticity and contractile function, and reduces skeletal muscle oxidative capacity and mitochondrial function [27–29]. In addition, according to Dunmore et al., participants with high G-Gap had lower activity of the desugarase fructosamine-3-kinase (FN3K) and were associated with higher levels of AGEs [30]. AGEs induce an inflammatory response by binding to their receptor RAGE, activating signaling pathways that promote the production of inflammatory cytokines, such as tumor necrosis factor-alpha (TNF-α) and interleukin-6 (IL-6), thus contributing to chronic inflammation [31–34]. Additionally, the accumulation of AGEs increases the generation of free radicals, leading to intracellular redox imbalance and subsequent cellular damage [35–37]. Inflammation and oxidative stress are significant contributors to impaired CRF [38–40]. Therefore, the association between G-Gap and impaired CRF may be explained in part by inflammation and oxidative stress.

It is important to recognize that the calculation of G-Gap still relies on measured HbA1c, and despite using standardized calculation methods, it may still be subject to statistical dependency. Furthermore, the accurate calculation of G-Gap requires validation through larger population-based studies. Therefore, assessment in larger samples may be necessary to derive a more precise calculation equation and determine the safe range of G-Gap, ultimately facilitating its incorporation into clinical practice. In addition, this study has several other limitations. First, due to the cross-sectional design, we cannot establish causal relationships between glycation gap and impaired cardiorespiratory fitness. Longitudinal studies are needed to confirm the temporal sequence and causality. Second, although we adjusted for multiple potential confounders, residual confounding factors might still exist. Some unmeasured variables, such as detailed medication use and dietary patterns, could affect both glycation status and CRF. Finally, the study population was limited to US adults, potentially limiting the generalizability of our findings to other populations with different ethnic backgrounds or lifestyle patterns. Future prospective studies with more comprehensive measurements and diverse populations are warranted to address these limitations.

In conclusion, this study is the first to demonstrate a significant association between higher G-Gap values and an increased likelihood of impaired CRF, providing new evidence for using G-Gap to assess cardiovascular health risks. Furthermore, the stratified analysis results in diabetic subgroups and the association between higher G-Gap values and an increased likelihood of impaired CRF independent of HbA1c suggest that attention to G-Gap is equally important even in populations with normal HbA1c levels. For individuals with a high G-Gap, even when their HbA1c levels appear to be within the controlled range, their actual blood glucose control may be worse. Therefore, relying solely on HbA1c may lead to misinterpretation of an individual's glycemic status. For individuals with a high G-Gap, a more comprehensive assessment should be conducted through continuous glucose monitoring, evaluating blood glucose fluctuations and analyzing insulin sensitivity. Incorporating the G-Gap concept into clinical practice can help identify the risk of glucose-related health damage in individuals with normal HbA1c levels. Some studies have suggested that there may be a certain genetic susceptibility to G-Gap [41], and targeting FN3K may be a potential intervention route [42]. These findings contribute to a better understanding of the complex relationship between glucose metabolism and cardiopulmonary function and provide new insights for developing individualized prevention strategies.

## Conclusions

This study demonstrates an association between higher G-Gap values and an increased likelihood of impaired CRF.

## Supplementary Information

Supplementary Material 1.

## Data Availability

All data are available as publicly accessible datasets through NHANES. It is open and publicly accessible through the following link:https://wwwn.cdc.gov/nchs/nhanes/.
