# Supplementary material for: Association between glycation gap and impaired cardiorespiratory fitness: evidence from American adults
Source: BMC Cardiovasc Disord. 2025 Feb 20;25:119. doi: 10.1186/s12872-025-04578-y (PMC11840999; doi:10.1186/s12872-025-04578-y)
Supplement: Supplementary file 1 — Supplementary Material 1. [file 12872_2025_4578_MOESM1_ESM.docx]

**Table S1. Classification of the Exclusion Criteria in the NAHNES Cardiovascular Fitness Component.**

**Table S2. Definition of complication.**

**Table S3. Threshold effect analysis between G-Gap and impaired CRF.**

**Table S4. The association between G-Gap** **and** **CRF (weighted)**

**Table S1. Classification of the Exclusion Criteria in the NAHNES Cardiovascular Fitness Component.**

| **Exclusion Category** | **Specific Conditions** |
| --- | --- |
| Physical functioning limitations | - Difficulties in walking for a quarter mile - Difficulties in walking up 10 steps without resting - Difficulties in walking from one room to another on the same level - Difficulties in standing up from an armless straight chair - Having back or neck problems - Having fractures or injuries in bone or joint - Having health problem that requires the use of special equipment, such as a cane or wheelchair - Having a bone or joint problem that could be made worse by walking - Lose balance due to dizziness on a regular basis - Lose consciousness on a regular basis - Blind or with very poor eyesight - Diabetes affected eyes or had retinopathy - Having developmental problems - Having amputations of legs or feet other than toes - Weight exceeded equipment limitation (>350 lb) - Other specified physical limitations |
| **Cardiovascular conditions/symptoms** | - Had been diagnosed with congestive heart failure - Had been diagnosed with coronary heart disease - Had been diagnosed with angina - Had been diagnosed with myocardial infarction - Had been diagnosed with stroke - Self-reported heart problems - Self-reported stroke problems - Having a pacemaker or automatic defibrillator - Doctor had instructed to do only physical activity recommended by a doctor because of a heart condition - Feeling chest pain during physical activity - Had chest pain when not doing physical activity - Resting heart rate ≥ 100 beats/min - Resting systolic blood pressure ≥ 180 mmHg - Resting diastolic blood pressure ≥ 100 mmHg - Irregular heart beats: 3 or more dropped beats in 30 seconds |
| Lung/breathing conditions/symptoms | - Having to stop for breath when walking at own pace on level ground - Having to stop for breath after walking about 100 yards or after a few minutes on level ground - Having been awakened by trouble breathing or shortness of breath - Having breathing trouble during sleep relieved by sitting up - Having to sleep on 2 or more pillows to help breathe - Self-reported lung or breathing problems - Had been diagnosed with emphysema |
| Asthma symptoms | - Had 12 or more attacks of wheezing or whistling during the past 12 months - Had wheezing severe enough to limit speech during the past 12 months |
| Medication exclusions | - Anti Arrhythmics   - Amiodarone (Cordarone)   - Bretylium (Bretylol)   - Disopyramide (Norpace)   - Encainide (Enkaid)   - Ethmozine (Moricizine)   - Flecanide (Tambocor)   - Lidocaine (Xylocaine, Xylocard)   - Metoprolol Succinate (Toprol-XL)   - Mexiletine(Mexitil) Moricizine (Ethmozine)   - Posicor (Mibefradil)   - Procainamide (Pronestyl, Procan SR)   - Propafenone (Rhythmol)   - Quinidine (Quinora, Quinalan, Cardioquin, Quinidex, Quinaglute)   - Tocainide (Tonocard) - Beta Blockers   - Acebutolol (Sectral)   - Atenolol (Tenormin)   - Betagan   - Betaxolol (Kerlone)   - Bisoprolol (Zebeta)   - Carteolol (Cartrol)   - Carvedilol (Coreg)   - Esmolol (Brevibloc)   - Labetalol (Normodyne)   - Levobunolol   - Metoprolol Tartrate (Lopressor)   - Nadolol (Corgard)   - Oxprenolol (Trasicor, Slow Trasicor)   - Penbutolol (Levatol)   - Pindolol (Visken)   - Propranolol (Inderal)   - Sotolol (Betapace)   - Timolol (Blocadren)   - Trandate - Beta Blockers/Diuretic Combinations   - Corzide   - Inderide   - Lopressor Hydrochlorothiazide   - Tenorectic   - Timolide   - Ziac - Calcium Channel-Blockers   - Amlodipine (Norvasc)   - Bepridil (Vascor)   - Diltiazem (Cardizem, Dilacor, Tiazac)   - Felodipine (Plendil)   - Isradipine (Dyna Circ)   - Norvasc (Amlodipine)   - Nicardipine (Cardene)   - Nifedipine (Procardia, Adalat)   - Nimodipine (Nimotop)   - Nisoldipine (Sular)   - Tiazak (Diltiazem HCl)   - Verapamil (Covera, Verelan, Calan, Isoptin) - CNS Stimulant   - Ma Huang (ephedrine) - Digitalis - Digoxin (Lanoxin) - Eye Drops/ Beta Blockers   - Betagen Eye Drops   - Betoptic Eye Drops   - Levobunolol Eye Drops   - Metipranolol (Optipranolol)   - Timoptic Eye Drops - Nitrates and Nitroglycerin   - Isosorbide Dinitrate (Isordil, Diltrate)   - Isosorbide Mononitrate (Ismo, Monoket)   - Nitroglycerin, Translingual (Nitrostat, Nitrolingual Spray)   - Nitroglycerin, Transmucosal (Nitrogard)   - Nitroglycerin, Topical (Nitrol, Nitro-Bid, Transderm Nitro, Nitro-Dur II, Nitrodisc, Minitran, Deponit, Nitroderm)   - Nitroglycerin, Sustained Release (Nitrong, Nitrocine, Nitroglyn)   - Pentaerythritol Tetranitrate (Cardilate) - Ephedra Based Weight Loss Medication   - Dymetadrine Xtreme   - Extreme Ripped Force   - Metabolife   - Metabolift   - Phentermine   - Pro-Ripped Ephedra   - Ripped Fuel   - Stacker   - Ultra Ripped |
| Other specified reasons | - Had been hospitalized for specified reasons in the past 3 months (see the CV Fitness Procedures Manual, Appendix D for details) - Doctor recommended not to participate in sports or other activities due to a health condition - Other safety concerns specified by the participant - Other safety concerns identified by MEC physician or staff |

**Table S2. Definition of complication.**

| Diabetes mellitus | Participants who met any of the following criteria were considered to have DM:   1. was told by doctor that had DM. 2. glycohemoglobin HbA1c (%) >= 6.5. 3. fasting glucose (mmol/l) >= 7.0. 4. random blood glucose (mmol/l) >= 11.1. 5. two-hour oral glucose tolerance test blood glucose (mmol/l) >= 11.1. 6. Use of diabetes medication or insulin. |
| --- | --- |
| Hypertension | Participants who met any of the following criteria were considered to have hypertension:   1. Systolic blood pressure ≥130mmHg or/and diastolic blood pressure ≥80mmHg. 2. Had been told by a doctor that had hypertension, 3. Antihypertensive drugs are currently being used |
| CKD | Participants who met any of the following criteria were considered to have CKD:  1. eGFR < 60 mL/min/ 1.73m^2^.  2.uACR > 30 mg/g. |
| Hyperlipidemia | Participants who met any of the following criteria were considered to have Hyperlipidemia:  1. Triglycerides ≥ 150 mg/dL.  2. Total cholesterol ≥ 200 mg/dL.  3. low-density lipoprotein ≥ 130 mg/dL.  4. high density lipoprotein≤ 40 mg/dL for men, and ≤ 50 mg/dL for women.  5. Take lipid-lowering drugs |
| Obesity | Participants with a BMI of 30 or more were considered obese |

**Table S3. Threshold effect analysis between G-Gap and impaired CRF.**

| **Model** | **OR (95% CI)** | **P-value** |
| --- | --- | --- |
| Model 1 Fitting model by standard linear regression | 1.414(1.007,1.984) | 0.046 |
| Model 2 Fitting model by two-piecewise linear regression |  |  |
| Infection point | -0.588 |  |
| <0.143 | 1.414(1.007,1.984) | 0.046 |
| >0.143 | 1.469(1.009,2.137) | 0.045 |
| P for likelihood ratio test |  | 0.340 |

Adjusted by age, gender, race, HB, WBC, PLT, Obesity, DM, Hypertension, Hyperlipidemia.

**Table S4. The association between G-Gap** **and** **CRF (weighted)**

|  | Model 1 | | | Model 2 | | | Model 3 | | | |  |
| --- | --- | --- | --- | --- | --- | --- | --- | --- | --- | --- | --- |
|  | OR (95%CI) | | *P*-value | | OR (95%CI) | *P*-value | | OR (95%CI) | *P*-value | |  |
|  | |  |  | |  |  | |  |  |  |  |
| G-Gap<0 | | Ref |  | | Ref |  | | Ref |  |  |  |
| G-Gap>0 | | 1.49(1.16,1.92) | 0.002 | | 1.71(1.32,2.20) | <0.001 | | 1.41(1.06,1.86) | 0.018 |  |  |

Model 1: Unadjusted.

Model 2: Adjusted by age, race, gender.

Model 3: Adjusted by age, race, gender, HB, WBC, PLT, Obesity, DM, Hypertension, Hyperlipidemia.
